# Recruit-aged adults may preferentially weight task goals over deleterious cost functions during short duration loaded and imposed gait tasks

**DOI:** 10.1038/s41598-023-31972-3

**Published:** 2023-03-25

**Authors:** Kellen T. Krajewski, Camille C. Johnson, Nizam U. Ahamed, Gavin L. Moir, Qi Mi, Shawn D. Flanagan, William J. Anderst, Chris Connaboy

**Affiliations:** 1 Department of Sports Medicine and Nutrition, University of Pittsburgh, Pittsburgh, PA USA; 2 Biodynamics Laboratory, Department of Orthopaedic Surgery, University of Pittsburgh, Pittsburgh, PA USA; 3 Exercise Science Department, East Stroudsburg University, East Stroudsburg, PA USA; 4 Center for Lower Extremity Ambulatory Research, Rosalind Franklin University of Medicine and Science, North Chicago, IL USA

**Keywords:** Musculoskeletal system, Occupational health

## Abstract

Optimal motor control that is stable and adaptable to perturbation is reflected in the temporal arrangement and regulation of gait variability. Load carriage and forced-marching are common military relevant perturbations to gait that have been implicated in the high incidence of musculoskeletal injuries in military populations. We investigated the interactive effects of load magnitude and locomotion pattern on motor variability, stride regulation and spatiotemporal complexity during gait in recruit-aged adults. We further investigated the influences of sex and task duration. Healthy adults executed trials of running and forced-marching with and without loads at 10% above their gait transition velocity. Spatiotemporal parameters were analyzed using a goal equivalent manifold approach. With load and forced-marching, individuals used a greater array of motor solutions to execute the task goal (maintain velocity). Stride-to-stride regulation became stricter as the task progressed. Participants exhibited optimal spatiotemporal complexity with significant but not meaningful differences between sexes. With the introduction of load carriage and forced-marching, individuals relied on a strategy that maximizes and regulates motor solutions that achieve the task goal of velocity specifically but compete with other task functions. The appended cost penalties may have deleterious effects during prolonged execution, potentially increasing the risk of musculoskeletal injuries.

## Introduction

Load carriage is a major component of military occupational tasks in combat-oriented roles implicated as a significant source of ‘noncombat’ musculoskeletal injuries (MSI)^1–3^. Different load magnitudes and locomotion patterns such as running (i.e., natural) or forced-marching (walking at a velocity beyond their gait transition velocity [GTV] where one would naturally jog [i.e., imposed]) are common conditions of military occupational gait tasks. However, much remains unclear as to how variability is distributed and regulated in response to ‘military relevant’ perturbations for longer durations. Even during unperturbed steady-state gait, considerable stride-to-stride variability is observed^4^. Healthy populations modulate their distribution and intertrial variability dynamics based on its relevance to the execution of a task goal, such as maintaining a specific velocity^5^ or throwing a frisbee to hit a specific target^6^. Likewise, healthy populations exhibit long-range correlational structure to their variability over time often referred to as spatiotemporal complexity, whereas neurologically impaired populations do not^7–9^. The discrimination between healthy and impaired populations for these measures indicates their potential utility as a marker of risk^4^. Preliminary research indicates low magnitude load carriage negatively alters the distribution of variability and decreases spatiotemporal complexity in healthy women during one-minute bouts of ambulation suggesting a potential link to observed MSI in military settings^4^. Employing a goal equivalent manifold (GEM) method, to provide a computational and conceptual framework, integrating geometric and nonlinear methods, enables the contextualization of any observed variability, potentially elucidating control strategies of the individual during gait^10^.

Gait is a complex motor task and the human locomotor system must incorporate many subsystems that operate on different timescales, evolve over time (dynamical) and exhibit nonlinear (chaotic) behavior^11–14^. Due to the numerous subsystems and their vast degrees of freedom, every aspect cannot be unilaterally controlled^15^. Consequently, individuals regulate behavior based on a goal manifold which represents an array of solutions that successfully execute a task based on a subconsciously defined goal^16^. For example, when walking on a treadmill at a specific velocity, a reasonably assumed goal is the maintenance of the treadmill velocity as to not fall off the treadmill and individuals will more strictly control stride variations that fail to achieve the treadmill velocity^10,17^. The goal manifold is represented in a multidimensional state-space (unitless representation of motor solution workspace) containing: (i) synergistic movement solutions^6,18^, (ii) multisensory information^19–23^, (iii) task/goal manifolds^10^ and (iv) cost function expense gradients (i.e., metabolic efficiency, energy dampening, stability, etc.) ^24–29^. Thus, state-space is dynamic and represented based on the individual’s perception. Within the state-space construct various attractor states are formed that provide the locomotor system ranges of behaviors to utilize (family of solutions). Not all attractor states are optimal, nor is state-space always accurately/appropriately represented to achieve optimal task performance outcomes, especially if the task is novel^20,23,30–35^. Nonetheless, attractor states provide the locomotor system the opportunity to leverage variability with minimal control effort, thus less emphasis on controlling every parameter and only regulating movement errors that interfere with the task goal^15^.

State-space is subdivided into null space (i.e., solutions that achieve the intended goal) and the task space (i.e., solutions that do not achieve the intended goal). Movements tangential to a goal manifold are null space variability (δ_T_) and those perpendicular are task space variability (δ_P_)^17,36,37^. Therefore, the ratio of tangential to perpendicular variability (relative variability)^16,17,36–38^ contextualizes motor variability with ratios greater than 1 indicating an individual leveraging their motor solution capacity in the null space effectively executing the task goal^39^. Indeed, healthy populations can have large spatiotemporal parameter variability (e.g., standard deviation), but will still exhibit more null space variability compared to task space variability^5^. Amputees have been observed with greater null space variability compared to task space when walking with a passive prosthetic^40^. However, at median ranges of gait velocities (~ 1.0 m/s), the amputated limb exhibits less task space variability compared to slower and higher gait velocities (U shape relationship)^40^ suggesting impaired modulation of task space variability at the edges of the individual’s preferred gait velocities. Women jogging and forced-marching with an external load of 45% of body weight (+ 45%BW) decreases relative variability ~ 21%^4^. Thus, perturbations can affect the distribution of null space and task space variability, especially as tasks are more physically challenging relative to the individual’s capabilities^4,40^, but it is unknown how heavier (> 45%) ‘military relevant’ loads (up to 60 kg^41^) may affect this. Additionally, these measures are just a geometric representation of variability and do not provide any temporal context and therefore provides limited information regarding movement regulation/control^10^.

Assessing the movement regulation (i.e., control of stride-to-stride fluctuations) of each subspace can reveal an individual’s ability to effectively mitigate movement errors without overloading the locomotor system^10,42^. While low fall risk elderly populations demonstrate a greater magnitude of variability, they regulate stride-to-stride variations the same as younger healthy adults^5^. As speed of locomotion increases, healthy individuals regulate stride-to-stride variations more strictly, correcting errors much quicker^37^. External load has similar effects as speed, with stride-to-stride control increasing for null and task space variability with load magnitudes up to + 45%BW, but this has only been observed during very short duration gait tasks (e.g., 1 min)^4^. Interestingly, as individuals increase neuromuscular fatigue they alter biomechanics to accommodate the task, but variations are still distributed and regulated the same regardless of fatigue status^43^. However, this phenomena has only been observed within an upper limb pushing task^43^ and remains unclear how ambulating with load for increased duration could affect these measures. Furthermore, stride-to-stride regulation of null space and task space variability only provides information about the short-term temporal correlation and not necessarily the long-term correlation often referred to as complexity which requires significantly longer trials^44^.

A system exhibiting ‘complexity’ is critically self-organized, meaning the independent subsystems act in concert to produce an emergent behavior^45^. Signals such as a stride length time-series that are complex exhibit temporal behavior where an interval in the present is influenced by an interval in the remote past^46–48^; thus, demonstrating long-range dependence/long-term memory^49^. This property is reflected in1/f or pink noise structure (α = 1.0) of the signal and is considered optimal as evidenced by healthy individuals exhibiting pink noise (i.e., complexity) in their spatiotemporal parameter time-series whereas neurologically impaired individuals exhibit white noise (i.e., stochasticity)^8,50,51^. Our previous work^4^ observed decreased spatiotemporal complexity as load magnitude increased and with the use of a forced-marching gait, in contrast to the natural locomotion pattern of running, which exhibited ideal spatiotemporal complexity (α ≈ 1.0) in women. Further investigation is warranted as it is unclear how the locomotor system would adjust to prolonged task execution, given the consequences of loaded gait tasks, which are routinely performed over extended periods of time. Also, extending the task duration would enable an increase in confidence and efficacy of the spatiotemporal complexity analysis results. Lastly, the previous sample was all women, raising the question; does sex result in a dimorphic locomotor response to military relevant perturbations?

Therefore, the primary purpose of the present study was to determine the interactive effects of load magnitude and locomotion pattern on motor variability, stride regulation and spatiotemporal complexity during prolonged gait tasks in recruit-aged adults. The secondary purpose was to examine the role of sex on motor variability, stride regulation and spatiotemporal complexity. Lastly, the tertiary purpose was to determine if motor variability and stride regulation was altered with time (increased task duration). It was hypothesized (**H**), that as load increases the use of forced-marching will constrain the locomotor system reflected by the reduction in relative variability (**H1**). Furthermore, increases in load and utilization of forced-marching would lead to stricter regulation strategies (**H2**). Spatiotemporal complexity was hypothesized to decrease as load increases and during the execution of the forced-marching locomotion pattern (**H3**), thus confirming results of prior research^4^. Additionally, due to anthropometric and physiological differences between sexes it was hypothesized that women will exhibit lower relative variability and less spatiotemporal complexity compared to men (**H4**). Lastly, it was hypothesized that as time progresses relative variability will further decrease as fatigue will begin to impact the number of motor solutions available (**H5**).

## Results

### Subject characteristics

Twenty-six individuals participated; however, six subjects (4 women and 2 men) were removed from the analysis due to data loss regarding the stride length or inability to perform protocol. Thus, the sample analyzed was n = 20 (men = 11, women = 9; see Table 1). There were no significant differences between men and women for age, trial velocities, or body mass index (BMI). Men were significantly taller (*p < *0.001) and heavier (*p = *0.004) than the women.

**Table 1:** Participant characteristics (mean ± SD).

Variable	Total (n = 20)	Men (n = 11)	Women (n = 9)
Age	26.0 ± 4.4	24.7 ± 4.6	27.6 ± 3.7
Height (m)	1.73 ± 0.1	1.79 ± 0.06*	1.65 ± 0.07
Weight (kg)	71.85 ± 16.12	79.07 ± 14.16*	63.02 ± 14.40
Body Mass Index (BMI)	23.9 ± 3.5	24.6 ± 3.7	23.0 ± 3.1
External load (kg)			
+ 45%BW	31.72 ± 7.41	35.20 ± 6.20*	27.46 ± 6.72
+ 55%BW	38.88 ± 8.79	42.97 ± 7.71*	33.87 ± 7.61
Velocity (m/s)			
BW	1.71 ± 0.21	1.73 ± 0.22	1.67 ± 0.20
+ 45%BW	1.66 ± 0.20	1.59 ± 0.10	1.74 ± 0.25
+ 55%BW	1.55 ± 0.22	1.60 ± 0.22	1.48 ± 0.21

* Men significantly (*p* ≤ 0.05) greater than women.

### Rating of perceived exertion (RPE)

**Figure 1:**
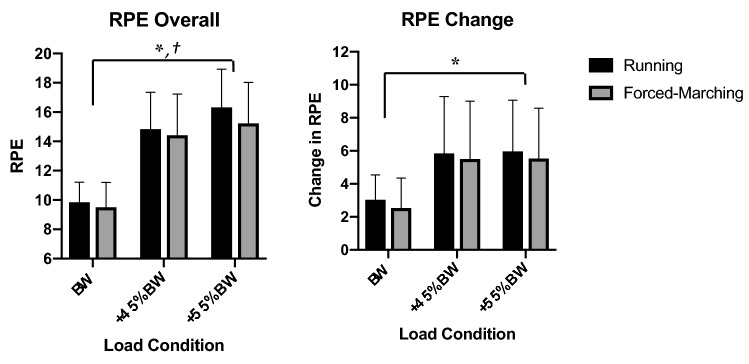
Ratings of Perceived Exertion (RPE). RPE = Borg scale (6–20); RPE Overall represent RPE of the entire trial; Change in RPE calculated as the final RPE value subtracting the first RPE value. * = Significant main effect of load; † = Significant main effect of locomotion*.*

Refer to Fig. 1 for RPE values (segregated by the total sample, men and women) which were collected as an indirect measure of metabolic effort. As load increased, overall RPE increased independent of locomotion pattern (*p < *0.001, η^2^_*p = *_0.79). Moreover, running resulted in a greater overall RPE compared to forced-marching independent of load condition (*p = *0.02, η^2^_*p = *_0.22). Likewise, when assessing change in RPE from minute 0 to minute 10, the addition of load carriage resulted in a larger change in RPE independent of locomotion pattern (*p < *0.001, η^2^_*p = *_0.42). There was no effect of sex on overall RPE or change in RPE.

### Spatiotemporal parameters

Refer to Table 2 for spatiotemporal parameter estimates during each condition respectively. When assessing the entire sample, mean stride length significantly decreased (~ 7%) as load magnitude increased for forced-marching only (*p = *0.006, η^2^_*p = *_0.44). Additionally, forced-marching had ~ 22% longer strides than running across all load conditions: BW (*p < *0.001, η^2^_*p = *_0.96); + 45%BW (*p < *0.001, η^2^_*p = *_0.90); and + 55%BW (*p < *0.001, η^2^_*p = *_0.95). When analyzing the between group effect of sex, men had longer strides than women for running (*p = *0.03, η^2^_*p = *_0.26) and forced-marching (*p < *0.001, η^2^_*p = *_0.54).

**Table 2:** Spatiotemporal parameter means and variance.

Load	Locomotion	Variable	Total	Men	Women
Mean					
BW	Running	Stride length	1.33 ± 0.14	1.37 ± 0.13	1.27 ± 0.14
		Stride time	0.76 ± 0.05	0.79 ± 0.05	0.76 ± 0.05
		Stride speed	1.71 ± 0.21	1.74 ± 0.22	1.67 ± 0.20
	Forced-marching	Stride length	1.65 ± 0.14	1.72 ± 0.12	1.56 ± 0.11
		Stride time	0.97 ± 0.08	1.00 ± 0.09	0.94 ± 0.06
		Stride speed	1.71 ± 0.21	1.73 ± 0.22	1.67 ± 0.20
+ 45%BW	Running	Stride length	1.32 ± 0.12	1.31 ± 0.09	1.34 ± 0.15
		Stride time	0.80 ± 0.06	0.82 ± 0.05	0.77 ± 0.06
		Stride speed	1.66 ± 0.20	1.59 ± 0.10	1.75 ± 0.26
	Forced-marching	Stride length	1.58 ± 0.09	1.61 ± 0.08	1.54 ± 0.08
		Stride time	0.96 ± 0.10	1.02 ± 0.06	0.89 ± 0.10
		Stride speed	1.66 ± 0.20	1.59 ± 0.10	1.75 ± 0.26
+ 55%BW	Running	Stride length	1.25 ± 0.15	1.31 ± 0.11	1.17 ± 0.17
		Stride time	0.81 ± 0.06	0.83 ± 0.06	0.79 ± 0.06
		Stride speed	1.55 ± 0.22	1.60 ± 0.22	1.48 ± 0.21
	Forced-Marching	Stride length	1.52 ± 0.13	1.59 ± 0.07	1.43 ± 0.14
		Stride time	0.99 ± 0.10	1.01 ± 0.11	0.97 ± 0.08
		Stride speed	1.55 ± 0.22	1.60 ± 0.22	1.49 ± 0.21
Variance					
BW	Running	Stride length	0.034 ± 0.010	0.038 ± 0.009	0.031 ± 0.010
		Stride time	0.016 ± 0.007	0.017 ± 0.007	0.015 ± 0.007
		Stride speed	0.040 ± 0.010	0.044 ± 0.012	0.036 ± 0.005
	Forced-marching	Stride length	0.026 ± 0.009	0.028 ± 0.010	0.024 ± 0.006
		Stride time	0.016 ± 0.005	0.016 ± 0.004	0.015 ± 0.006
		Stride speed	0.023 ± 0.010	0.027 ± 0.012	0.019 ± 0.004
+ 45%BW	Running	Stride length	0.038 ± 0.017	0.041 ± 0.016	0.034 ± 0.018
		Stride time	0.019 ± 0.012	0.022 ± 0.014	0.016 ± 0.008
		Stride speed	0.042 ± 0.014	0.045 ± 0.017	0.038 ± 0.010
	Forced-marching	Stride length	0.037 ± 0.048	0.029 ± 0.007	0.046 ± 0.073
		Stride time	0.021 ± 0.021	0.019 ± 0.005	0.024 ± 0.032
		Stride speed	0.021 ± 0.021	0.019 ± 0.005	0.024 ± 0.032
+ 55%BW	Running	Stride length	0.036 ± 0.011	0.038 ± 0.012	0.034 ± 0.009
		Stride time	0.020 ± 0.009	0.021 ± 0.010	0.020 ± 0.009
		Stride speed	0.036 ± 0.007	0.039 ± 0.007	0.033 ± 0.004
	Forced-marching	Stride length	0.035 ± 0.019	0.038 ± 0.020	0.032 ± 0.017
		Stride time	0.024 ± 0.013	0.024 ± 0.009	0.024 ± 0.017
		Stride Speed	0.026 ± 0.010	0.030 ± 0.012	0.022 ± 0.005

mean ± standard deviation.

Stride length in m; stride time in seconds; stride speed in m/s.

For mean stride time, forced-marching had significantly longer stride times compared to running (*p < *0.001, η^2^_*p = *_0.96). Men had longer stride times than women for forced-marching only (*p = *0.03, η^2^_*p = *_0.24). There were no significant effects of load magnitude or locomotion pattern for mean stride speed. Additionally, there were no significant effects of sex on mean stride speed.

Refer to Table 2 for all mean and standard deviations of each spatiotemporal parameter’s variance at each condition respectively. For stride length variance there were no significant effects. As load increased, stride time variance significantly increased independent of locomotion pattern (*p = *0.02, η^2^_*p = *_0.26). For stride speed variance, running exhibited greater stride speed variance than forced-marching (*p < *0.001, η^2^_*p = *_0.77). There were no significant between-groups effects of sex on spatiotemporal parameter variance.

**Table 3:** Relative Variability.

Load	Locomotion	Portion	Total	Men	Women
BW	Running	Total	1.56 ± 0.24	1.53 ± 0.18	1.58 ± 0.30
		Beginning	1.52 ± 0.16	1.52 ± 0.14	1.53 ± 0.18
		End	1.49 ± 0.31	1.42 ± 0.15	1.56 ± 0.41
	Forced-Marching	Total	1.91 ± 0.45	1.73 ± 0.50	2.09 ± 0.32
		Beginning	1.95 ± 0.45	1.71 ± 0.45	2.20 ± 0.29
		End	1.73 ± 0.52	1.57 ± 0.58	1.89 ± 0.42
+ 45%BW	Running	Total	1.66 ± 0.25	1.72 ± 0.24	1.60 ± 0.25
		Beginning	1.62 ± 0.36	1.76 ± 0.41	1.46 ± 0.22
		End	1.56 ± 0.38	1.60 ± 0.40	1.51 ± 0.36
	Forced-Marching	Total	2.00 ± 0.37	1.95 ± 0.37	2.07 ± 0.39
		Beginning	1.85 ± 0.41	1.91 ± 0.33	1.79 ± 0.51
		End	1.87 ± 0.39	1.83 ± 0.38	1.92 ± 0.41
+ 55%BW	Running	Total	1.90 ± 0.60	1.79 ± 0.58	2.01 ± 0.62
		Beginning	1.66 ± 0.25	1.67 ± 0.25	1.65 ± 0.27
		End	1.83 ± 0.73	1.78 ± 0.72	1.88 ± 0.78
	Forced-Marching	Total	2.29 ± 0.88	2.04 ± 0.42	2.55 ± 1.15
		Beginning	2.18 ± 0.63	2.03 ± 0.45	2.33 ± 0.77
		End	2.09 ± 1.01	1.92 ± 0.46	2.27 ± 1.37

mean ± standard deviation.

BW = Body weight (no load); + 45%BW = An additional 45% of BW; + 55%BW = An additional 55% of BW.

Total = Entire trial; Beginning = First 30% of the trial only; End = Last 30% of the trial only.

### Relative variability

**Figure 2:**
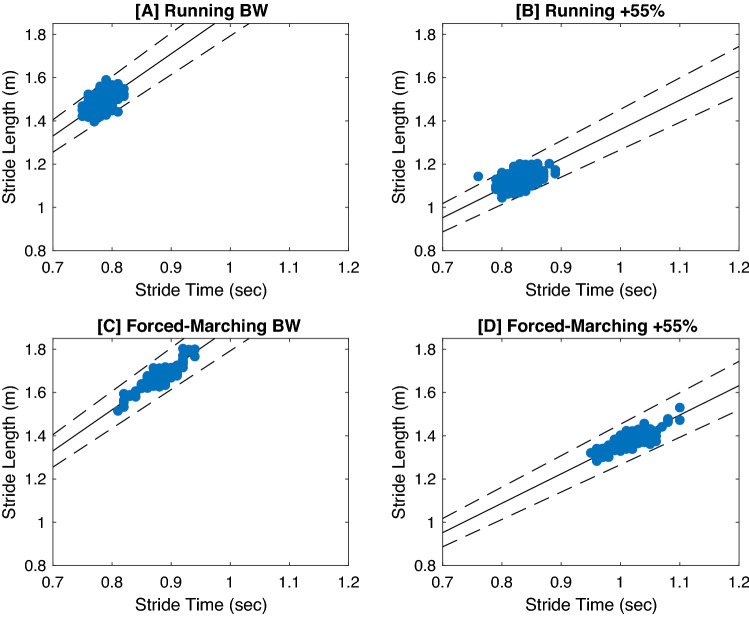
Exemplar GEM Plots. Exemplar GEM plots of a single female participant during the unloaded (BW) and + 55%BW load conditions. The solid line represents the goal manifold where each point on the line is a stride time and stride length combination that achieves the trial velocity. The dotted lines above and below the goal manifold are 5% error bars. Both the natural locomotion conditions of running, (**A**) and (**B**), exhibit a wider spread perpendicular to the goal manifold and have relative variabilities of 1.39 and 1.71 for this participant respectively. Moreover, some of the variations are outside of the ± 5% error bars (**B**). Conversely, the imposed locomotion condition of forced-marching, (**C**) and (**D**), exhibits a wider spread along the goal manifold and have relative variabilities of 2.09 and 2.36 respectively. While forced-marching may result in more stride variations that achieve the task goal of maintaining trial velocity, the larger spread indicates a greater range of coordinative patterns, some of which may be deleterious in nature.

GEM decomposition was performed on each participant separately and presented as an average, see Fig. 2 for exemplar GEM plot. Additionally, refer to Table 3 for all GEM related outcomes. When analyzing relative variability of the entire trial length, as load magnitude increased, relative variability increased independent of locomotion pattern (*p = *0.01, η^2^_*p = *_0.29). Additionally, forced-marching had greater relative variability compared to running (*p < *0.001, η^2^_*p = *_0.43).

Following the assessment of relative variability of the entire trial the influence of time on relative variability (first 30% of the trial vs. the final 30% of the trial) was analyzed. There were no significant effects of time on relative variability. Exploratory analysis to assess the possibility that relative variability might differ between men and women did not indicate the presence of sex-specific effects.

### Subspace variance

Refer to Table 4 for variance tangential and perpendicular (subspace variance) to the goal manifold for each condition. Tangential variability (null space) significantly increased with increases in load magnitude (*p = *0.004, η^2^_*p = *_0.46). Moreover, forced-marching had greater tangential variability than running (*p < *0.001, η^2^_*p = *_0.49). By contrast, perpendicular variability (task space) decreased significantly with increases in load magnitude (*p = *0.003, η^2^_*p = *_0.48) and running exhibited greater perpendicular variability than forced-marching (*p < *0.001, η^2^_*p = *_0.50). Additionally, tangential variability was greater than perpendicular variability for each load condition regardless of locomotion pattern: BW (*p < *0.01, η^2^_*p = *_0.93); + 45%BW (*p < *0.001, η^2^_*p = *_0.97); and + 55%BW (*p < *0.001, η^2^_*p = *_0.96).

**Table 4:** Subspace variability.

Load	Locomotion	Variable	Portion	Total	Men	Women
BW	Running	Tangential	Total	1.18 ± 0.05	1.18 ± 0.02	1.18 ± 0.06
			Beginning	1.08 ± 0.12	1.07 ± 0.15	1.10 ± 0.09
			End	1.16 ± 0.20	1.13 ± 0.11	1.20 ± 0.26
		Perpendicular	Total	0.77 ± 0.08	0.78 ± 0.06	0.76 ± 0.10
			Beginning	0.72 ± 0.11	0.71 ± 0.12	0.73 ± 0.11
			End	0.79 ± 0.11	0.80 ± 0.11	0.78 ± 0.11
	Forced-Marching	Tangential	Total	1.23 ± 0.08	1.20 ± 0.09	1.27 ± 0.04
			Beginning	1.17 ± 0.16	1.12 ± 0.16	1.23 ± 0.14
			End	1.16 ± 0.18	1.11 ± 0.20	1.21 ± 0.16
		Perpendicular	Total	0.67 ± 0.13	0.73 ± 0.15	0.62 ± 0.07
			Beginning	0.62 ± 0.12	0.68 ± 0.13	0.57 ± 0.07
			End	0.71 ± 0.18	0.77 ± 0.22	0.66 ± 0.10
+ 45%BW	Running	Tangential	Total	1.21 ± 0.05	1.22 ± 0.04	1.19 ± 0.05
			Beginning	1.05 ± 0.21	1.08 ± 0.22	1.00 ± 0.21
			End	1.19 ± 0.34	1.20 ± 0.33	1.18 ± 0.38
		Perpendicular	Total	0.73 ± 0.08	0.72 ± 0.07	0.76 ± 0.08
			Beginning	0.67 ± 0.17	0.65 ± 0.18	0.70 ± 0.15
			End	0.76 ± 0.12	0.76 ± 0.13	0.78 ± 0.10
	Forced-Marching	Tangential	Total	1.26 ± 0.05	1.25 ± 0.05	1.26 ± 0.05
			Beginning	1.14 ± 0.22	1.21 ± 0.09	1.05 ± 0.30
			End	1.21 ± 0.21	1.20 ± 0.15	1.22 ± 0.28
		Perpendicular	Total	0.64 ± 0.09	0.66 ± 0.10	0.63 ± 0.09
			Beginning	0.63 ± 0.15	0.65 ± 0.10	0.60 ± 0.20
			End	0.66 ± 0.10	0.67 ± 0.09	0.65 ± 0.12
+ 55%BW	Running	Tangential	Total	1.23 ± 0.07	1.21 ± 0.07	1.25 ± 0.06
			Beginning	1.01 ± 0.18	1.04 ± 0.15	0.97 ± 0.20
			End	1.26 ± 0.27	1.30 ± 0.21	1.21 ± 0.33
		Perpendicular	Total	0.69 ± 0.13	0.71 ± 0.13	0.66 ± 0.13
			Beginning	0.62 ± 0.14	0.64 ± 0.14	0.60 ± 0.14
			End	0.73 ± 0.14	0.77 ± 0.13	0.68 ± 0.13
	Forced-Marching	Tangential	Total	1.28 ± 0.05	1.26 ± 0.05	1.29 ± 0.05
			Beginning	1.12 ± 0.23	1.13 ± 0.24	1.10 ± 0.23
			End	1.20 ± 0.26	1.28 ± 0.31	1.12 ± 0.19
		Perpendicular	Total	0.60 ± 0.12	0.64 ± 0.10	0.56 ± 0.13
			Beginning	0.54 ± 0.12	0.56 ± 0.10	0.51 ± 0.13
			End	0.63 ± 0.16	0.69 ± 0.15	0.57 ± 0.17

mean ± standard deviation.

Tangential represents ‘null space’ variability; Perpendicular represents ‘task space’ variability.

Lastly, the effects of time (first 30% of the trial versus the final 30% of the trial) were assessed for each subspace variability (tangential and perpendicular) separately. There were no significant effects of time on tangential variability. However, perpendicular variability was greater in the final 30% of the trial compared to the beginning 30% of the trial (*p < *0.001, η^2^_*p = *_0.53).

### Stride regulation

Refer to Table 5 for alpha coefficients of GEM coordinate time series tangential and perpendicular to the goal manifold for each condition. When assessing stride regulation of tangential variability forced-marching had greater persistence (less strict stride-to-stride control) than running independent of load condition (*p = *0.05, η^2^_*p = *_0.19). When assessing stride regulation of perpendicular variability, as load magnitude increased, stride-to-stride control increased, evidenced by decreased persistence (*p = *0.04, η^2^_*p = *_0.16). Additionally, forced-marching had less persistence (more strict control) compared to running regardless of load condition (*p = *0.02, η^2^_*p = *_0.24).

**Table 5:** Stride-to-stride regulation.

Load	Locomotion	Variable	Portion	Total	Men	Women
BW	Running	Tangential α	Total	0.78 ± 0.12	0.77 ± 0.10	0.79 ± 0.14
			Beginning	0.77 ± 0.15	0.72 ± 0.11	0.81 ± 0.18
			End	0.69 ± 0.17	0.63 ± 0.13	0.75 ± 0.19
		Perpendicular α	Total	0.65 ± 0.09	0.66 ± 0.09	0.65 ± 0.09
			Beginning	0.61 ± 0.10	0.60 ± 0.10	0.62 ± 0.10
			End	0.56 ± 0.14	0.54 ± 0.15	0.58 ± 0.14
	Forced-Marching	Tangential α	Total	0.85 ± 0.10	0.86 ± 0.12	0.84 ± 0.09
			Beginning	0.82 ± 0.12	0.82 ± 0.13	0.83 ± 0.11
			End	0.73 ± 0.12	0.67 ± 0.12	0.80 ± 0.10
		Perpendicular α	Total	0.60 ± 0.12	0.61 ± 0.11	0.59 ± 0.13
			Beginning	0.56 ± 0.12	0.56 ± 0.14	0.56 ± 0.10
			End	0.55 ± 0.10	0.55 ± 0.10	0.55 ± 0.12
+ 45%BW	Running	Tangential α	Total	0.82 ± 0.10	0.81 ± 0.11	0.82 ± 0.08
			Beginning	0.77 ± 0.17	0.78 ± 0.15	0.76 ± 0.20
			End	0.69 ± 0.13	0.71 ± 0.16	0.66 ± 0.09
		Perpendicular α	Total	0.63 ± 0.10	0.64 ± 0.13	0.62 ± 0.06
			Beginning	0.55 ± 0.18	0.53 ± 0.16	0.58 ± 0.21
			End	0.51 ± 0.17	0.54 ± 0.21	0.47 ± 0.11
	Forced-Marching	Tangential α	Total	0.86 ± 0.11	0.80 ± 0.08	0.92 ± 0.12
			Beginning	0.86 ± 0.16	0.86 ± 0.11	0.87 ± 0.21
			End	0.77 ± 0.19	0.74 ± 0.10	0.80 ± 0.26
		Perpendicular α	Total	0.58 ± 0.10	0.53 ± 0.09	0.64 ± 0.08
			Beginning	0.57 ± 0.10	0.56 ± 0.10	0.59 ± 0.10
			End	0.50 ± 0.12	0.45 ± 0.07	0.56 ± 0.13
+ 55%BW	Running	Tangential α	Total	0.83 ± 0.11	0.79 ± 0.09	0.88 ± 0.12
			Beginning	0.77 ± 0.14	0.74 ± 0.11	0.80 ± 0.18
			End	0.68 ± 0.20	0.67 ± 0.17	0.69 ± 0.24
		Perpendicular α	Total	0.61 ± 0.10	0.59 ± 0.10	0.62 ± 0.10
			Beginning	0.51 ± 0.15	0.51 ± 0.13	0.51 ± 0.18
			End	0.46 ± 0.16	0.49 ± 0.12	0.43 ± 0.19
	Forced-Marching	Tangential α	Total	0.87 ± 0.13	0.78 ± 0.10	0.95 ± 0.09
			Beginning	0.86 ± 0.16	0.82 ± 0.18	0.89 ± 0.14
			End	0.77 ± 0.17	0.73 ± 0.18	0.82 ± 0.14
		Perpendicular α	Total	0.54 ± 0.10	0.51 ± 0.11	0.57 ± 0.09
			Beginning	0.53 ± 0.11	0.55 ± 0.15	0.51 ± 0.06
			End	0.47 ± 0.12	0.51 ± 0.13	0.43 ± 0.08

*mean* ± *standard deviation.*

α = alpha coefficient derived from detrended fluctuation analysis;

BW = Body weight (no load); + 45%BW = An additional 45% of BW; + 55%BW = An additional 55% of BW;

Total = Entire trial; Beginning = First 30% of the trial only; End = Last 30% of the trial only;

α = 0.5 represents stochastic (random) control; α < 0.5 represents anti-persistent regulation (strict control); α > 0.5 represents persistent behavior (looser/weaker control); α ≥ 1.5 represents over regularity (no control).

When assessing the influence of time for tangential variability regulation, the first 30% of the trial exhibited greater persistence compared to the final 30% of the trial regardless of condition (*p < *0.001, η^2^_*p = *_0.52). Similarly, for perpendicular variability regulation, the first 30% of trial had more persistence compared to the final 30% of the trial regardless of trial condition (*p = *0.01, η^2^_*p = *_0.28).

When assessing the effect of sex on the entire trial tangential variability regulation, women exhibited less strict control (more persistence) as load magnitude increased (*p = *0.02, η^2^_*p = *_0.39), whereas stride regulation remained unchanged across load conditions for men (*p = *0.67, η^2^_*p = *_0.05). At the + 55%BW load condition only, men exhibited greater stride-to-stride control (less persistence) compared to women regardless of locomotion pattern (*p = *0.004, η^2^_*p = *_0.38). For the entire trial perpendicular variability regulation there were no significant effects of sex.

### Spatiotemporal complexity

Refer to Table 6 for spatiotemporal parameter time-series alpha coefficients (α) [complexity]. For stride length complexity, forced-marching exhibited greater complexity than running independent of load condition (*p < *0.001, η^2^_*p = *_0.50). Further, complexity increased as load magnitude increased for women only (*p = *0.02, η^2^_*p = *_0.38); whereas stride length complexity remained unchanged across load conditions for men (*p = *0.87, η^2^_*p = *_0.02). Additionally, at the + 55%BW load condition only, women exhibited greater stride length complexity than men (*p = *0.009, η^2^_*p = *_0.32).

**Table 6:** Spatiotemporal Alpha Coefficients (Complexity).

Load	Locomotion	Variable	Total	Men	Women*‡*
BW (No additional load)	Running	SL	0.68 ± 0.10	0.68 ± 0.10	0.68 ± 0.14
		ST	0.79 ± 0.11	0.79 ± 0.11	0.80 ± 0.12
		SS	0.35 ± 0.10	0.36 ± 0.13	0.34 ± 0.04
	Forced-Marching	SL	0.80 ± 0.11*	0.80 ± 0.14	0.81 ± 0.08
		ST	0.82 ± 0.10	0.81 ± 0.10	0.83 ± 0.11
		SS	0.41 ± 0.11*	0.42 ± 0.11	0.41 ± 0.11
+ 45%BW	Running	SL	0.75 ± 0.10	0.75 ± 0.10	0.76 ± 0.11
		ST	0.84 ± 0.11	0.84 ± 0.13	0.84 ± 0.09
		SS	0.37 ± 0.11	0.37 ± 0.14	0.37 ± 0.06
	Forced-Marching	SL	0.82 ± 0.11*	0.76 ± 0.08	0.88 ± 0.12
		ST	0.79 ± 0.10	0.74 ± 0.09	0.86 ± 0.07*†*
		SS	0.42 ± 0.08*	0.40 ± 0.08	0.44 ± 0.07
+ 55%BW	Running	SL	0.76 ± 0.11	0.73 ± 0.09	0.80 ± 0.13*†*
		ST	0.84 ± 0.10	0.80 ± 0.09	0.88 ± 0.10*†*
		SS	0.29 ± 0.04	0.30 ± 0.03	0.29 ± 0.04
	Forced-Marching	SL	0.83 ± 0.14*	0.74 ± 0.11	0.92 ± 0.09*†*
		ST	0.81 ± 0.14	0.71 ± 0.10	0.90 ± 0.11*†*
		SS	0.38 ± 0.06*	0.39 ± 0.04	0.38 ± 0.08

mean ± standard deviation.

SL = Stride Length; ST = Stride Time; SS = Stride Speed.

BW = Body weight (No additional load); + 45%BW = Plus an additional 45% of BW; + 55%BW = Plus an additional 55% of BW.

α = .5 represents stochastic (white noise); α < 0.5 represents anti-persistence; 0.75 < α < 1.3 represents persistent behavior (optimal complexity); α = 1 equal persistent behavior exhibiting power law scaling (pink noise); α ≥ 1.5 represents over regularity (brown noise).

*Significantly greater than running.

†Significantly greater than men; ‡Significant simple main effect of load for SL and SS (complexity increasing with load).

For stride time complexity there were no significant effects of load magnitude or locomotion. However, women exhibited greater complexity than men at both loaded conditions regardless of locomotion pattern; + 45%BW (*p = *0.05, η^2^_*p = *_0.17) and + 55%BW (*p < *0.001, η^2^_*p = *_0.41).

For stride speed complexity, there was a significant main effect of load (*p = *0.002, η^2^_*p = *_0.27), with + 45%BW being greater (*p = *0.004) than + 55%BW. Additionally, forced-marching stride speed complexity was greater than running independent of load condition (*p < *0.001, η^2^_*p = *_0.48). However, there were no effects of sex on stride speed complexity.

## Discussion

We assessed the interactive effects of load magnitude and locomotion pattern on motor variability, stride regulation and spatiotemporal complexity during prolonged gait tasks in recruit-aged adults and potential interactions with sex and task duration. While the results failed to support the stated experimental hypotheses (**H1–H5**), they do however support findings presented by published researchers^5,17,37^. Specifically, healthy, recreationally fit recruit-aged (18–35 years) men and women adopt a goal manifold relevant control strategy during *treadmill-based* gait tasks^5,17,37^. Despite the introduction of novel/unfamiliar perturbations and constraints of load carriage and forced-marching, individuals from the present sample exhibited optimal spatiotemporal complexity (stride time and stride length α = 0.75–1.00 [Table 6]), relative variability > 1.0 (Table 3), leveraged null space variability (greater tangential variability compared to perpendicular variability) (Table 4), minimally controlled null space motor solutions (α≈1.0) and tightly controlled task space motor solutions (α ≈ 0.5) (Table 5) with limited meaningful differences between sexes. These findings suggest that this sample population execute state-space exploratory behavior primarily to execute the task goal, but the greater relative variability during the loaded forced-marching conditions may be pursued at the expense of deleterious cost functions and potentially increase MSI risk (see Fig. 2). Thus, careful examination of the confluence of findings, may however reveal important discriminations, which need to be considered further, especially when generalizing from the dimensionally limited confines of the laboratory to a more dimensionally rich, real-world setting.

Previous load carriage research ^4^, observed relative variability and spatiotemporal complexity decreased with increases in load magnitude and forced-marching. In contrast to the aforementioned investigation ^4^, we observed that as the load magnitude increased, relative variability increased by ~ 20% from BW to + 55%BW load conditions and spatiotemporal complexity remained optimal across conditions (failing to support H1 and H3). Similar to previous findings, forced-marching demonstrated ~ 25% more relative variability than running regardless of load condition^4^. Indeed, relative variability values during the running at BW trial (Table 3) were consistent with those observed in healthy younger and elderly adult populations ambulating (running and walking) at preferred speeds^5,17,37^. These conflicting results could be due to two factors: (i) trials in the present investigation were considerably longer (~ 10 min versus 1.5 min), and (ii) the previous investigation required participants to walk for 30 s at a velocity 10% below their GTV and then transitioned to 10% above GTV and remaining at that velocity for one minute (only this final minute was analyzed), whereas the velocity was ramped up to the trial velocity and then the trial began in the present study. Due to the shorter trial duration a reduced number of strides (< 512) were collected potentially yielding false positives for detrended fluctuation analysis (DFA). However, the findings of the present study can be stated with greater veracity, given that stride counts exceeded 512 strides (1265 ± 295 strides across all trials)^44^. Additionally, the 30 s period below GTV potentially entrenched the individual in an attractor state optimized for a true walking condition (as they were inexperienced with load carriage). Thus, once perturbed into the forced-marching velocity, an individual may have been reluctant to engage in state-space exploratory behavior (observed as lower relative variability as load magnitude increased^4^).

Task skill and variability have been considered inversely related, however greater variability of task execution can result in reduced outcome variability (i.e., less deviations from the trial velocity in the present investigation)^52^. The caveat to the previous statement being that greater variability is observed in the null space specifically and task space variability is reduced during redundant/cyclical tasks (i.e., walking)^6,53–55^. In the present investigation, participants increased null space variability (tangential) ~ 4% and decreased task space variability (perpendicular) ~ 12% as the task became more ‘difficult’ with increasing load magnitude and forced-marching. Moreover, participants more strictly controlled task space variability and allowed more persistence (looser control) in the null space. Thus, variability serves a multitude of purposes including task exploration (adaptation/learning/skill acquisition) and flexibility/adaptability to perturbation (i.e., degeneracy)^52^. Most likely however, there is an optimal amount of variability (in terms of the ratio between null space and task space variability [i.e., relative variability]) as too little or too much could be detrimental to performance^56^.

The observed larger relative variability (> 1.9 [Table 3]) in conjunction with the optimal spatiotemporal complexity (Table 6) and less strict control of null space (Table 4) during forced-marching and loaded conditions may indicate state-space exploratory behavior. It should be noted that this sample was unfamiliar with loaded forced-marching, similar to military recruit populations. During the early stages of a novel task execution, variability is considered task solution space exploration^57^. This ‘exploration’ can be thought of as an experimentation with various movement solutions (stride length and time combinations in the present investigation) to discover an optimal movement pattern^57^. Initial stages of learning a novel/unfamiliar task are associated with more random and larger magnitude excursions in motor variability as a means to intentionally explore the task solution space (especially when null space is unknown, such as during novel tasks)^57^ and perceptual-motor workspace (cost landscape) which can resolve cost estimates and identify optimal dimensions^53,58–60^. Specifically, the observed behavior was reflected by the looser control of stride-to-stride variations for null and task space variability (Table 5) during the first 30% of the trial compared to the final 30%. Therefore, deviations were allowed to persist in a certain direction longer. This behavior suggests that the individuals were *exploring* their task solution space early on to identify an optimal family of solutions (i.e., a group of stride variations that achieve trial velocity). Important to exploration optimization, these deviations are not always random, but exhibit long-range correlation (both stride length and stride time timeseries exhibited α = 0.75–1.0 in the present investigation [Table 6])^57,61,62^. During the ‘exploratory’ phase of variability, viable task solutions are ‘formed’ and representative of the solution task space^57^. At the later stages of learning/exploration there is a transition to smaller scale searches of the task solution space (i.e., more refined experimentation)^57^. Future research should investigate multiple trials of the same condition to confirm if this is indeed state-space exploration and determine if individuals learn from previous bouts and adapt their behavior accordingly.

While state-space exploratory behavior may explain observed variability and appear beneficial to the adaptation of new perturbations/constraints (e.g., load carriage and forced-marching), it can also impede adaptation as well. An important component of motor behavior optimization is the need to identify optimal strategies quickly^58^. Given the vast number of degrees of freedom of the locomotor system, not only in terms of joint actions but motor units/neural circuitry as well, the system must efficiently determine the objective function of a task and adapt behavior to evolving constraints (i.e., influence of fatigue)^58,63,64^. In the present investigation, relative variability remained unchanged from first 30% to the final 30% of the task (contrary to H5) and *both* null space and task space variability regulation became stricter (~ 11% more control [Table 5]), potentially indicating that participants were never able to effectively constrain their motor variability to an optimal range of motor solutions. Because the sample is novice with respect to the task, they have little experience to draw from and direct their searches to *preferred* or *experienced* attractor states even if inappropriate for the given motor task^65–70^. This latter point was highlighted by the greater task space variability (Table 4) observed during running compared to forced-marching (regardless of load condition), suggesting individuals executed more strides that failed to achieve trial velocity as they were reverting to preferred frequencies. Secondly, the lack of experience potentially inhibits the locomotor system organization due to the competition (or inappropriate weighting) of various cost functions. As a consequence of the challenge(s) occurring in response to the novelty of the perturbation(s) (increased load and gait type), the locomotor system explores the perceptual-motor landscape, over state-space, in the attempt to establish an optimal attractor state (as indicated by relative variability significantly increasing with increases in load magnitude and forced-marching) in relation to cost function (re)weighting as it evolves throughout the task.

State-space exploration was likely organized predominantly to address the task goal (achieving/maintaining trial velocity) during the loaded and forced-marching conditions neglecting other important cost functions, owing to the relatively short duration of the task and the constrained environment in which it was performed (treadmill). In the present investigation, participants perceived exertion (RPE) of the loaded conditions changed significantly more than the unloaded conditions (6 ± 3 vs 3 ± 2 [Fig. 1]) despite all experimental trials being performed at the same relative velocities suggesting the use of motor solutions that were not all conducive to mechanical/metabolic efficiency. Likewise, stride speed exhibited anti-persistent behavior (mean α = 0.37 ± 0.08 for all conditions) indicating that any deviations in velocity from the trial velocity in one direction were quickly corrected in the opposite direction to return to the trial velocity. In general participants considered the loaded conditions more difficult than the unloaded as evidenced by the overall RPEs (unloaded = 10 ± 1 versus loaded = 16 ± 3, where the maximum RPE is 20). Therefore, keeping pace with the trial velocity to stay on the treadmill dominates most of the locomotor system’s attention to cost function weighting (i.e., locomotor system dimensionally constrains the motor problem to engage in *successful* task execution of maintaining trial velocity).

Dimensional constraining of state-space or task goal overweighting yielded optimal results in the laboratory setting but may ultimately lead to greater MSI risk in natural dimensionally rich environments. While spatiotemporal complexity (mean α = 0.77 ± 0.11 and α = 0.82 ± 0.11 for stride length and stride time, respectively) reflected long-range correlation and minimal control of the individual stride parameters, these parameters do not necessarily encapsulate complexity regarding other cost functions. Utilizing a forced-marching locomotion pattern exhibited ~ 22% longer strides on average compared to running regardless of load magnitude. Longer strides being performed during forced-marching compared to running is likely a compensation to achieve the trial velocity (task goal) as forced-marching eliminates a flight phase^71–73^. However, forced-marching may disrupt stability (balance) and lead to more extended joints of the lower extremity at the moment of impact (heel-strike) impeding the ability to attenuate force^74^. Likewise, the limited joint excursions at the knee during loaded forced marching shifts mechanical work proximal to the hip in women^75^. Moreover, in women, forced-marching with loads up to 45% of BW significantly increases the knee abduction moment which has been linked to knee osteoarthritis^76^. Therefore, individuals utilize a large range of motor solutions (greater relative variability) that *benefit achieving the task goal specifically* (i.e., maintaining trial velocity) but may enact a physical toll, when considering other, more deleterious cost functions such as kinetic (i.e., greater mechanical stress) or balance (i.e., more likely to trip with additional perturbations)^26–29^. In shorter durations (~ 10 min) and dimensionally constrained settings (laboratory) executing these motor solutions with larger cost penalties in the kinetic or balance domain may be tolerable, but in military settings load carriage tasks can persist for hours. Relative variability > 2 (as observed during forced-marching + 55%BW condition) may explain the high incidence of MSI during load carriage related activities, especially if this behavior is maintained during the prolonged (i.e., hours) activity^1–3^. Further investigation is warranted to determine if motor specific training can improve recruit population locomotor function during loaded gait tasks.

There are some limitations to consider in this investigation that need to be acknowledged. Firstly, relatively small sample sizes may explain the lack of clinically meaningful differences for sex-specific comparisons. In military settings, absolute loads are used regardless of stature and differences in motor behavior may be observed between sexes due to women being smaller and therefore standardized load carriage representing a greater percentage magnitude of their bodyweight. While standardized combat boots were provided to control for the effects of footwear on kinematics, they may have been a source of observed changes in motor behavior. Combat boots often result in pain and blisters, especially those less ‘broken in’^77^. It is therefore possible that participants modulated their stride-to-stride variability to ameliorate foot pain rather than adhere to the task goal. Lastly, some aspects of the investigated sample represented a military recruit population (i.e., age, stature, relative fitness, and lack of load carriage/combat boot experience); the results of this investigation may not generalize to other adult populations (e.g., lower fitness levels). Indeed 17.4% of the military is classified as obese^78^ whereas none of the participants in the current sample were classified as obese (by BMI standards). A fitter sample was determined as a more practical starting point to assess temporal variability with load carriage. Many analysis techniques require large numbers of consecutive data points to return valid results (i.e., DFA needs at least 512)^44,79^. Fitter individuals were more likely to successfully execute the full ten-minute trial (or at minimum enough time to be included in the analysis). Therefore, results of the present study can only be generalized to men and women on the healthier/fitter end of the spectrum.

In conclusion, for healthy, recreationally fit recruit-aged men and women, in a controlled, treadmill-based setting (dimensionally constrained), the locomotor system can adapt (evidence by long-range correlations) to perturbations of load magnitude up to + 55%BW and forced-marching for short periods (~ 10 min). Likewise, with the introduced perturbations, the locomotor system can expand the null space, while constraining and tightly controlling the task space (reduced perpendicular variability and α ≈ 0.5 for perpendicular coordinate time series) to achieve a task goal of maintaining a specific velocity. Interestingly, sex failed to have any meaningful effects on locomotor system function. Importantly, the coalesced representation of the findings suggest that this sample population execute state-space exploratory behavior primarily to execute the task goal. The greater relative variability during the loaded forced-marching conditions suggest that behavior is pursued even at the expense of accruing penalties of more deleterious cost functions, which would be unsustainable in the ecological representation of the task in a military setting. Furthermore, excessive variability, even null space variability that is optimally regulated, may portend the risk of MSI and explain the high incidence in military settings.

## Methods

### Participants

Twenty-six (13F, 13 M) healthy and physically active recruit-aged (18–35 years) adults participated in this investigation (See Table 1 for all participant characteristics). Physically active was operationally defined as engaging in moderate to intense exercise a minimum of three days a week and ability to run on a treadmill at 2.68 m/s for ten minutes. Potential participants were excluded if they had a musculoskeletal injury (i.e., precluding from physical activity or requiring modified physical activity) in the past six months, neurological disorder or were pregnant. All participants were informed of the potential risks of the investigation prior to the obtainment of written informed consent. The investigation was approved by the University of Pittsburgh’s internal review board and all experimental methods were performed in accordance with all relevant guidelines/regulations including the Declaration of Helsinki.

### Materials and procedures

Participants attended a single session where they completed an equipment familiarization, GTV determination trials, and ten-minute trials of running and forced-marching with no load (BW), an additional 45% of their bodyweight (+ 45%BW) and an additional 55% of their bodyweight (+ 55%BW). Spatiotemporal parameters were determined via three-dimensional (3D) kinematic data captured at 100 Hz using 12 infrared cameras (Vicon Motion Systems, Oxford, UK). Kinetic data were captured via an instrumented split-belt treadmill at 1000 Hz with a velocity resolution of 0.01 m/s. All participants were provided and appropriately fitted with combat boots (Speed 3.0 Boot, 5.11 Tactical, Irvine, CA) to reduce effects of footwear on lower extremity kinematics. Additionally, thick heavy-duty moisture wicking socks (Athletic Crew Socks, ONKE, US) were provided to reduce likelihood of blisters. Loads were added using a combination of a single size plate carrier (Testudo Gen 2, Armored Republic, Phoenix, AZ) and a small weight-vest (Short Plus Style Vest, MIR, US) [loads less than 37 kg] or large weight-vest (EZ-Vest, Kensui Fitness, Sheridan, CO) [loads greater than 37 kg]. Weight-vests were placed on top of the plate carrier and secured tightly to reduce extraneous movement of the load. Weight was distributed 40% anteriorly and 60% posteriorly to closely mimic military relevant loading^80^ and control for the effects to center of mass (COM) displacement^81^.

Before performing experimental trials, participants executed a ten-minute familiarization trial unloaded. The familiarization consisted of five minutes of walking at a brisk pace (identified as a rating of perceived exertion [RPE] effort between 8 and 10 on the 20-point Borg scale). Following the first five minutes of walking participants transitioned directly into a light jog (velocity that achieved a 10–12 RPE rated effort). Familiarization was performed on the split-belt treadmill in provided combat boots. During this period adjustments to boot size were made if necessary. Following the familiarization participants, were prepared for data collection with the placement of retroreflective markers. Markers were placed on the calcaneus and the 1^st^ and 5^th^ metatarsophalangeal (MTP) joints to create foot segments to capture stride length and stride time spatiotemporal parameters.

Prior to each experimental load condition (BW, + 45%BW and + 55%BW), the GTV was determined utilizing a ramped treadmill protocol accelerating at 0.05 m/s^2^^76^. Mean GTV were established by conducting three trials of the ramped protocol before the performance of each load condition. Experimental trials were performed at 10% above mean GTV for that specific load condition. Participants were allowed a brief rest between the GTV determination trials and the start of the experimental trial. Data collection started once participants had reached the necessary trial velocity and prolonged for ten minutes (or until the participant could no longer continue). Trials concluded early if participants verbally indicated they could no longer continue or if they were about to fall off the treadmill.

Participants were instructed to adopt a ‘natural’ and comfortable locomotion pattern for the run trials and to maintain a walking gait irrespective of the treadmill velocity for the forced-marching trials. A member of the research team was always present near the treadmill to ensure participant safety and that they maintained a walking gait during the forced-marching trials. Additionally, RPE was obtained as a surrogate measure of metabolic effort every two minutes starting at minute 0 (when the treadmill reached experimental velocity) by the present research team member. Overall RPE was collected following the completion of the experimental trial. Lastly, RPE change was calculated as the difference between final RPE and first RPE. Load conditions were randomized first and then locomotion patterns within the load condition were randomized to control for order effects. Participants were given ten minutes rest between each trial to minimize the effects of fatigue.

### Data reduction

All data was processed in Visual 3D (C-Motion, Germantown, MD, USA) and analyzed with custom MATLAB (Mathworks, Inc., Natick, MA) scripts. Data was smoothed with a low-pass Butterworth filter with a cutoff frequency of 6 Hz and 25 Hz for kinematic and kinetic data, respectively^4^. Strides were identified by vertical ground reaction forces (vGRF) exceeding 50N for initial contact (heel-strike) and final contact point before vGRF dropped below 50N (toe-off). A stride was considered from heel-strike to ipsilateral heel-strike. Stride length (m) was calculated as the distance travelled from heel-strike to ipsilateral heel-strike. Stride time (s) was calculated as the time elapsed from heel-strike to ipsilateral heel-strike. Stride speed (m/s) was calculated as stride length divided by stride time. Spatiotemporal parameter time-series were composed into three components: 1) the entire trial (ten minutes or total time the participant completed); 2) beginning phase (the first 30% of the trial); and 3) final phase (the last 30% of the trial). The mean and standard deviation were calculated for each spatiotemporal parameter for the entire trial only.

Once spatiotemporal parameter time-series were prepared, GEM decomposition (see Dingwell et al*.*^17^ for detail on the GEM data reduction method) was utilized to generate tangential (δ_T_) and perpendicular (δ_P_) coordinates for each participant separately. The standard deviation (σ) of δ_T_ and δ_P_ coordinate time-series were determined for each load and locomotion condition. Relative variability was calculated as the ratio between σδ_T_/ σδ_P_. Scaling exponents (α) were computed from δ_T_ and δ_P_ time-series through detrended fluctuation analysis (DFA) (see Delignières et al.^44^ for detailed methods). Interpretation of scaling exponents for δ_T_ and δ_P_ time-series were: α < 0.5 represents anti-persistence (alteration in one direction more likely followed by an alteration in opposite direction); α > 0.5 represents statistical persistence (alteration in one direction more likely followed by an alteration in same direction); and α = 0.5 represents uncorrelated (alteration in one direction has same likelihood of being followed by alteration in either direction)^5,17^. GEM related outcomes (relative variability, σδ_T_, σδ_P_, δ_T_α, and δ_P_α) were calculated for the entire trial, the beginning phase and the final phase.

Lastly, DFA was conducted on spatiotemporal parameter time-series (stride length, stride time and stride speed) to assess gait complexity, specifically long-range correlation (for detailed methods of DFA see Delignières et al*.*^44^). The smallest window size was 10 and the largest window equal to half the signal length with 16 window sizes between the smallest and largest, for a total of 18 separate window sizes, equally log spaced utilizing the method of Almurad and Delignières^82^. Evenly spaced window sizes (log spacing) yield up to 36% less variation from the true alpha of the signal when compared to arbitrarily chosen window sizes^82^. DFA was conducted on the entire trial only as time-series < 512 consecutive data points reduce the validity of alpha coefficients (α)^44^. Spatiotemporal parameter complexity outcomes were interpreted as α < 0.75 represents white noise (stochasticity); 0.75 < α < 1.2 represents pink noise, a balance between deterministic and stochastic processes; and α > 1.3 represents brown noise (over-regularity)^4,8^.

### Statistical analysis

Descriptive statistics (mean and SD) were reported for all outcome variables. To determine if there were significant differences between sexes (men vs. women), independent t-tests were conducted for age, height, weight, body mass index (BMI), trial velocities and trial loads separately. To contextualize metabolic intensity and gait characteristics of experimental trials, a two-way repeated measure analysis of variance (RMANOVA) for Load × Locomotion (3 × 2) was conducted separately on overall RPE, change in RPE and spatiotemporal parameter mean and variances. To address the primary purpose a two-way RMANOVA Load × Locomotion (3 × 2), was conducted on each GEM and gait complexity outcome separately. To further elucidate changes in relative variability, a Load × Locomotion × Subspace (σδ_T_ and σδ_P_) (3 × 2 × 2) mixed factor RMANOVA was conducted.

To determine the potential influence time had on motor variability and stride regulation, a Time (trial phase) × Locomotion × Load (2 × 2 × 3) mixed factor RMANOVA was conducted separately on relative variability, σδ_T_, σδ_P_, δ_T_α, and δ_P_α. Lastly, to address the secondary purpose, an exploratory analysis was conducted to examine the influence of the group factor of sex on all outcomes. Therefore, a three-way Sex × Locomotion × Load (2 × 2 × 3) mixed factor RMANOVA was conducted separately on each outcome.

For two-way RMANOVA (3 × 2), if interactions were significant, simple main effects were performed (paired t-tests for 2-level independent variables stratified by interactive factor, and RMANOVA for 3-level independent variables stratified by interactive factor). Post-hoc analysis using Bonferroni-corrected pairwise comparisons were conducted when necessary. If no significant interaction was observed, only main effects were analyzed. For mixed factor RMANOVAs (2 × 2 × 3), if a significant three-way interaction was observed, then simple main effects were assessed for each level of the interaction. If no significant three-way interaction was observed, then only two-way interactions of Load × Sex/Time/Direction and Locomotion × Sex/Time/Direction were examined. If no significant two-way interaction was observed, only the main effect of Sex/Time/Direction was examined.

For all RMANOVA, if sphericity was violated (indicated by a significant p value (*p* ≤ 0.05) of Mauchly’s tests of sphericity) then Greenhouse–Geisser adjusted values were reported (denoted by the degrees of freedom). For mixed factor RMANOVA Box’s M test and Mauchly’s sphericity were executed to test equality of covariance and ensure assumptions of sphericity are met. Partial eta squared (η^2^_p_) was calculated as a measure of effect size with magnitudes of effect interpreted as: 0.01–0.085 (small effect); 0.09–0.24 (moderate effect); and > 0.25 (large effect)^83^. Alpha level set at 0.05.

## Data Availability

Data is available upon reasonable request by contacting the corresponding author at kxk836@case.edu.
